# Pangenome analysis of *Bifidobacterium longum* and site-directed mutagenesis through by-pass of restriction-modification systems

**DOI:** 10.1186/s12864-015-1968-4

**Published:** 2015-10-21

**Authors:** A. O’Callaghan, F. Bottacini, M. O’Connell Motherway, D. van Sinderen

**Affiliations:** APC Microbiome Institute & School of Microbiology, University College Cork, Western Road, Cork, Ireland

**Keywords:** *Bifidobacterium longum*, Comparative genomics, Pan-genome, Probiotics, Restriction modification systems, Methylation

## Abstract

**Background:**

Bifidobacterial genome analysis has provided insights as to how these gut commensals adapt to and persist in the human GIT, while also revealing genetic diversity among members of a given bifidobacterial (sub)species. Bifidobacteria are notoriously recalcitrant to genetic modification, which prevents exploration of their genomic functions, including those that convey (human) health benefits.

**Methods:**

PacBio SMRT sequencing was used to determine the whole genome seqeunces of two *B. longum* subsp. *longum* strains. The *B. longum* pan-genome was computed using PGAP v1.2 and the core *B. longum* phylogenetic tree was constructed using a maximum-likelihood based approach in PhyML v3.0. *M.blmNCII* was cloned in *E. coli* and an internal fragment if *arfB*arfB was cloned into pORI19 for insertion mutagenesis.

**Results:**

In this study we present the complete genome sequences of two *Bifidobacterium longum* subsp. *longum* strains. Comparative analysis with thirty one publicly available *B. longum* genomes allowed the definition of the *B. longum* core and dispensable genomes. This analysis also highlighted differences in particular metabolic abilities between members of the *B. longum* subspecies *infantis, longum* and *suis*. Furthermore, phylogenetic analysis of the *B. longum* core genome indicated the existence of a novel subspecies. Methylome data, coupled to the analysis of restriction-modification systems, allowed us to substantially increase the genetic accessibility of *B. longum* subsp. *longum* NCIMB 8809 to a level that was shown to permit site-directed mutagenesis.

**Conclusions:**

Comparative genomic analysis of thirty three *B. longum* representatives revealed a closed pan-genome for this bifidobacterial species. Phylogenetic analysis of the *B. longum* core genome also provides evidence for a novel fifth *B. longum* subspecies. Finally, we improved genetic accessibility for the strain *B. longum* subsp. *longum* NCIMB 8809, which allowed the generation of a mutant of this strain.

**Electronic supplementary material:**

The online version of this article (doi:10.1186/s12864-015-1968-4) contains supplementary material, which is available to authorized users.

## Background

Bifidobacteria have been isolated from several ecological niches linked to the gastro intestinal tract (GIT) of animals, including the human GIT, where they represent prominent members of the gut microbiota [1, 2]. Bifidobacteria have attracted significant scientific and commercial interest due to their purported health-promoting or probiotic effects conferred to their (human) host, such as strengthening/maintenance of the intestinal barrier, modulation of the immune system, and pathogen exclusion [3, 4].

Fourty eight different bifidobacterial species are currently recognised, including nine subspecies, and of these only fifteen species are represented by fully assembled genome sequences, of which there are currently fifty four publicly available (June 2015, source: http://www.ncbi.nlm.nih.gov/Taxonomy/Browser/wwwtax.cgi). Multiple genome sequences are available for certain bifidobacteiral species allowing for pan-genome analysis, which is the total number of different genes encoded by a certain species [5]. Recent studies have deduced the pan-genome for *Bifidobacterium breve* [6] and *Bifidobacterium animalis* subsp. *lactis* [7], while another focussed on the genomic diversity of *Bifidobacterium adolescentis* [8]. In order to map the evolutionary development of the *Bifidobacterium* genus, an extensive comparative study was recently performed on individual representatives of 47 bifidobacterial (sub)species [9, 10]. One of these comparative studies suggests that bifidobacteria and their animal hosts co-evolved, and that this co-evolution was facilitated by both gene loss and acquisition events to allow for (sub)species-specific adaptations to a glycan-rich environment [9].

Functional genome analysis of bifidobacteria is important in order to understand how this species adapts to a particular niche. For example, more than 8 % of the annotated genes found in the genomes of *B. longum* and *B. breve* are predicted to encode proteins involved in the metabolism of complex plant-derived carbohydrates [11]. In contrast, the human genome is predicted to encode just seventeen enzymes involved in glycan catabolism [12, 13]. It is believed that this paucity is compensated by the metabolic abilities provided by the gut microbiota, including bifidobacteria, thus allowing the (human) host to (indirectly) digest complex polysaccharides that would otherwise be deemed non-digestible. It is therefore important to obtain an in depth understanding of carbohydrate utilisation by gut commensals and its impact on their host.

Despite the generally held view that bifidobacteria elicit positive health effects on their host, the underlying molecular mechanisms are as yet far from fully understood [4]. One of the key reasons for this knowledge gap is the difficulty in genetically modifying bifidobacteria, in part attributed to the presence of restriction-modification (R-M) systems [4, 14], which provide a powerful and natural defence for prokaryotic cells against invading foreign DNA, in particular bacteriophages [15]. R-M systems are currently classified into four well characterised types, I, II, III and IV, based on their co-factor requirements, protein composition, and target/cleavage sequence characteristics [16]. Type I R-M systems consist of three subunits that are responsible for methylation, specificity and restriction, respectively, and recognise asymmetric sequences that comprise of two components that are separated by a non-specific spacer. Type I R-M systems require *S-*Adenosylmethionine (AdoMet), ATP and Mg^2+^, typically methylate adenine residues and cut unmodified DNA at sites distal to the recognition sites [16]. Classical type II R-M systems recognise palindromic sequences of 4–8 bp in length and cut DNA into discrete fragments within or close to the recognition site [16]. The type II R-M methyltransferase (MTase) modifies adenosyl or cytosyl residues of a particular recognition sequence, which, when unmethylated, is recognised and cut by the corresponding restriction endonuclease (REase) [17, 18]. Type III R-M systems consists of two subunits that are responsible for DNA recognition and modification (Mod subunit), and DNA cleavage (Res subunit). Type III R-M systems recognise inversely orientated asymmetric DNA sequences, where the Res subunit cuts the DNA close to one of these recognition sites [19]. Finally, type IV restriction enzymes differ from those previously described as they recognise and cleave DNA only when the recognition site is methylated [18].

Independent studies in bifidobacteria have demonstrated that following modification of plasmid DNA, R-M systems can be by-passed, thereby resulting in a substantial increase in transformation efficiency and in some cases the successful generation of mutants [15, 20]. In a recent study, data gained from methylome analysis led to a moderate improvement in the transformation efficiency of the strain *Bifidobacterium animalis* subsp. *lactis* CNCM I-2494 [21]. However, overcoming R-M systems of a given species is but one tool of the expanding bifidobacterial genetic tool box, which now includes conjugation-based methods [22, 23], a temperature-sensitive plasmid [24] and a double-crossover, marker-less gene deletion system [25].

In this study we present the complete genome sequence for *B. longum* subsp. *longum* NCIMB 8809 and *B. longum* subsp. *longum* CCUG 30698, and associated methylome and R-M analyses. Comparative analysis of the genomes of these two strains with publicly available complete and incomplete *B. longum* genomes enabled us to explore the genomic diversity among members of the *B. longum* subspecies *longum, infantis* and *suis.* In addition, by exploiting methylome and genomic data analysis, we were able to assess the functionality of the R-M systems native to *B. longum* subsp. *longum* NCIMB 8809. This allowed us to improve the genetic accessibility of *B. longum* subsp. *longum* NCIMB 8809, permitting site-directed mutagenesis of this strain.

## Results and discussion

### General features of *B. longum* genomes

**Fig. 1:**
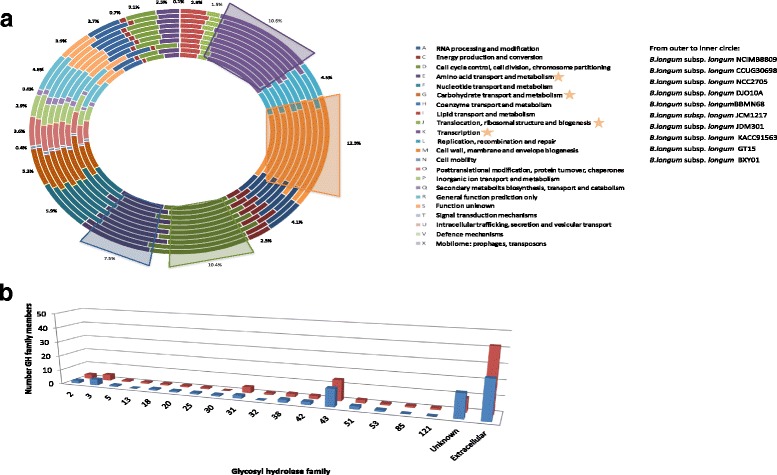
Comparative genomics of fully sequenced *B. longum* subsp. *longum* genomes. **a** Cluster of Orthologues (COG) classification of all ORFs from publicly available *B. longum* subsp. *longum* genomes. For each COG entry the average percentage of hits among *B. longum* subsp. *longum* has been indicated. The most abundant COG families are assigned to housekeeping functions and have been indicated. From the outer to inner circle: *B. longum* subsp. *longum* NCIMB 8809, *B. longum* subsp. *longum* CCUG 30698, *B. longum* subsp. *longum* NCC2705, *B. longum* subsp. *longum* DJO10A, *B. longum* subsp. *longum* BBMN68, *B. longum* subsp. *longum* JCM1217, *B. longum* subsp. *longum* JDM301, *B. longum* subsp. *longum* KACC91563, *B. longum* subsp. *longum* GT15 and *B. longum* subsp. *longum* BXY01. **b** The number of glycosyl hydrolase family members for *B. longum* subsp. *longum* NCIMB 8809 (blue) and *B. longum* subsp. *longum* CCUG 30698 (red). The number of predicted extracellular glycosyl hydrolases is indicated

The complete genome sequence was determined for two *B. longum* subsp. *longum* strains that had been isolated from infant faeces (*B. longum* subsp. *longum* NCIMB 8809) or a human adult intestine (*B. longum* subsp. *longum* CCUG 30698). Salient details of each of these genomes are presented in Table 1. The observed G + C% content of both *B. longum* genomes (60.1 % G + C% content for *B. longum* subsp. *longum* NCIMB 8809 and 60.22 G + C% content for *B. longum* subsp. *longum* CCUG 30698) is consistent with that reported for other bifidobacterial genomes [3]. The genome of *B. longum* subsp. *longum* CCUG 30698 contains a substantially higher number of tRNAs compared to most other sequenced *B. longum* strains, although a similarly high number of tRNA-encoding elements is present in the genome of strain *B. longum* subsp. *infantis* ATCC 15697 [26]. BLASTP analyses of deduced proteins of all identified ORFs in both *B. longum* genomes was performed against the Cluster of Orthologous Groups (COG) database and the obtained results show that a high percentage of predicted proteins is dedicated to general cellular housekeeping functions including amino acid transport and metabolism, and carbohydrate transport and metabolism (10.6 % in the case of *B. longum* subsp. *longum* NCIMB 8809 and 12.3 % in the case of *B. longum* subsp. *longum* CCUG 30698). These percentages are consistent with those previously observed for other bifidobacterial genomes [6, 27–29] (Fig. 1, panel a).

**Table 1:** *Bifiobacterium longum* general genome features

	*B. longum* subsp. *longum* NCIMB 8809	*B. longum* subsp. *longum* CCUG 30698	*B. longum* subsp. *longum* NCC2705	*B. longum* subsp. *longum* DJO10A
Isolated from	Nursling stool	Human adult intestine	Infant faeces	Young adult faeces
Genome Size	2.34	2.45	2.25	2.37
G + C content %	60.1	60.22	60.12	60.15
Number of identified genes	1872	1983	1727	1990
Percentage of genes functionally assigned	77 %	74 %	79 %	76 %
Prophage	1 (complete)	1	1	1
Episome	1	0	1	1
rRNA	3	2	4	4
tRNA	56	70	57	58
CRISPR	0	0	1	1

Further exploration of the sequence data revealed that both genomes encode a single homologous phosphoenolpyruvate-phosphotransferase system (PEP-PTS). This system exhibits 34 % identity across 99 % of the query sequence to a fructose-specific PEP-PTS system encoded by *B. breve* UCC2003 [30]. In relation to carbohydrate-active enzymes, 35 glycosyl hydrolases (GHs) were identified in *B. longum* subsp. *longum* NCIMB 8809, whereas *B. longum* subsp. *longum* CCUG 30698 is predicted to encode 40 GHs (Fig. 1, panel b). The *B. longum* subsp. *longum* NCIMB 8809 genome specifies GHs that belong to thirteen different GH families, while the *B. longum* subsp. *longum* CCUG 30698 genome is predicted to specify GHs that are from fifteen different GH families. Interestingly, both genomes are predicted to encode a high number of GHs that belong to the GH family 43. *B. longum* subsp. *longum* NCIMB 8809 is predicted to encode twelve members of GH family 43, seven of which are predicted to be extracellular (Fig. 1, panel b). *B. longum* subsp. *longum* CCUG 30698 on the other hand is predicted to encode fourteen GH43 family members of the, of which nine are predicted to be extracellular (Fig. 1, panel b). Many members of the GH43 family represent inverting enzymes active against long-chain carbohydrates (polysaccharides), which are typically constituents of plant cell walls and represent so-called non-digestible dietary fibers, such as arabinoxylan, arabinan, galactan and xylan [31]. Such predicted plant polysaccharide-degrading activities had previously been described for the *B. longum* subsp. *longum* NCC2705 genome, when it was reported that, according to COG functional classification, more than 8.5 % of the predicted proteins encoded by this strain were associated with carbohydrate metabolism and transport.

### Synteny and variability of *B. longum* subsp. *longum* genomes

To investigate the syntenic relationship between the two newly sequenced *B. longum* genomes, dotplot comparisons were constructed using *B. longum* subsp. *longum* NCC2705 as the reference genome. The resulting dotplots between the genomes of *B. longum* subsp. *longum* NCC2705 and *B. longum* subsp. *longum* NCIMB 8809 reveal a near continuous straight line indicating a high level of synteny (Additional file 1: Figure S1). The comparison between *B. longum* subsp. *longum* NCC2705 and *B. longum* subsp. *longum* CCUG 30698 reveals a break in genome synteny due to the presence of an apparent DNA inversion. This inversion concerns a 550,317 bp region on the *B. longum* subsp. *longum* CCUG 30698 genome. Examination of this CCUG 30698 region by PCR confirmed that this DNA inversion is genuine, and apparently stable, thus representing a distinctive feature of this genome (data not shown).

Various extracellular structures encoded by bifidobacteria have been associated with host colonisation and gut persistence, interaction with the host immune system [32–34], such as pili or fimbriae and surface-associated EPS (sEPS) [32, 34]. *B. longum* subsp. *longum* NCIMB 8809 and *B. longum* subsp. *longum* CCUG 30698 also contain genetic information for such extracellular structures (Table 2). The individual sEPS clusters identified in *B. longum* subsp. *longum* NCIMB 8809 and *B. longum* subsp. *longum* CCUG 30698 appear to lack one or more critical functions: the sEPS-specifying cluster in *B. longum* subsp. *longum* NCIMB 8809 encodes a single glycosyl transferase (GT) (a predicted priming GT), but does not contain a flippase- or other GT-encoding genes, while that of *B. longum* subsp. *longum* CCUG 30698 does not appear to encode a priming GT or polymerase (data not shown). These observations are consistent with the sedimenting phenotype of these two strains during planktonic growth (data not shown), which was also observed for a *B. breve* UCC2003-derived mutant carrying a deletion in the gene cluster responsible for sEPS production [35].

**Table 2:** *Bifidobacterium longum* variable regions that are indicated by their respective locus tags

Variable region	*B. longum* subsp. *longum* NCIMB 8809	*B. longum* subsp. *longum* CCUG 30698	*B. longum* subsp. *longum* NCC2705	*B. longum* subsp. *longum* DJO10A
Prophage 1a	B8809_1609 – B8809_1668	-	-	-
Prophage 1b	-	BBL306_1148_ BBL306_1177	-	-
Prophage 1c	-	-	-	BLD_1131 – BLD1161*
Prophage 1d	-	-	BL0367 – BL0386	-
Episome 1a	B8809_0936 – B8809_1001	-	-	-
Episome 1b	-	-	BL1458 – BL1495	-
Episome 1c	-	-	-	BLD_0337 – BLD_0395
CRISPR	-	-	-	BLD_1903 – BLD1904
EPS cluster 1a	B8809_0330 – B8809_0356	-	-	-
EPS cluster 1b	-	BBL306_0398 – BBL306_0424	-	-
EPS cluster 1c	-	-	BL0225 – BL0237	-
EPS cluster 1d	-	-	-	BLD_1565 – BLD_1579
EPS cluster 2a	B8809_1724 – B8809_1741	-	-	-
EPS cluster 2b	-	BBL306_1452 – BBL306_1466	-	-
EPS cluster 2c	-	-	BL0203 – BL0215	-
EPS cluster 2d	-	-	-	BLD_1565 – BLD_1579
R/M system 1	B8809_0606 – B8809_0607*	-	BL1473 – BL1474*	-
R/M system 2	B8809_0958	-	-	-
R/M system 3	B8809_1350 – B8809_1355*	BBL306_1749 – BBL306_1756 *	BL1778 – BL1785*	BLD_1954 – BLD_1962*
R/M system 4		BBL306_1025		
R/M system 5	-	BBL306_0230 – BBL306_0231	-	-
R/M system 6	-	BBL306_0910 – BBL306_0911	-	-
R/M system 7	-	-	BL0563 – BL0565	-
R/M system 8	-	-	-	BLD_1565 – BLD_1579
*pil* (Sortase dependent)	B8809_1829 – B8809_1831*	BBL306_1003 – BBL306_1005*	BL0674 – BL0676-*	BLD_1467 – BLD_1469*
*pil*	B8809_1607 – B8809_1608*	BBL306_1519 – BBL306_1520*	-	-
*pil* (Tad)	B8809_0107 – B8809_0112 and B8809_0717*	BBL306_0121 – BBL306_0126 and BBL306_0758*	BL0504 – BL0509 and BL0879*	BLD_0613 and BLD_1293 - BLD_1298*

Homologous genes are indicated within the table and are marked with a "*".

Bifidobacteria appear to be subject to regular phage attacks, events that are expected to contribute to variability within bifidobacterial genomes [36]. In light of this, searches for prophage-like elements revealed that *B. longum* subsp. *longum* NCIMB 8809 and *B. longum* subsp. *longum* CCUG 30698 harbor prophage-like elements (Table 2). The prophage-like element identified in *B. longum* subsp. *longum* NCIMB 8809 appears to be complete, whereas *B. longum* subsp. *longum* CCUG 30698 appears to harbor a single, apparently incomplete prophage-like element (due to the absence of genes that encode replication functions). Comparative analysis reveals that the prophage-like elements identified here differ from each other and that they are integrated at different locations. Although integrated at different positions within the genome, both prophage-like elements identified in *B. longum* subsp. *longum* NCIMB 8809 and *B. longum* subsp. *longum* CCUG 30698 are integrated in a tRNA^Gly^ gene. The genome of *B. longum* subsp. *longum* NCIMB 8809 also harbors a putative 25 kb episome integrated in a tRNA^Asn^ gene [37] (Table 2 and Additional file 2: Table S3).

### Pan-genome analysis

In order to evaluate the total gene repertoire of currently sequenced representatives of the *B. longum* species, we applied a pan-genome analysis pipeline [38]. A total of thirty three *B. longum* genomes (complete and incomplete genome sequences) were included in this analysis. Where relevant, genome sequences were subject to re-annotation using the same annotation procedure described previously so as to ensure that all annotations and identified ORFs were comparable (See Materials and Methods section).

**Fig. 2:**
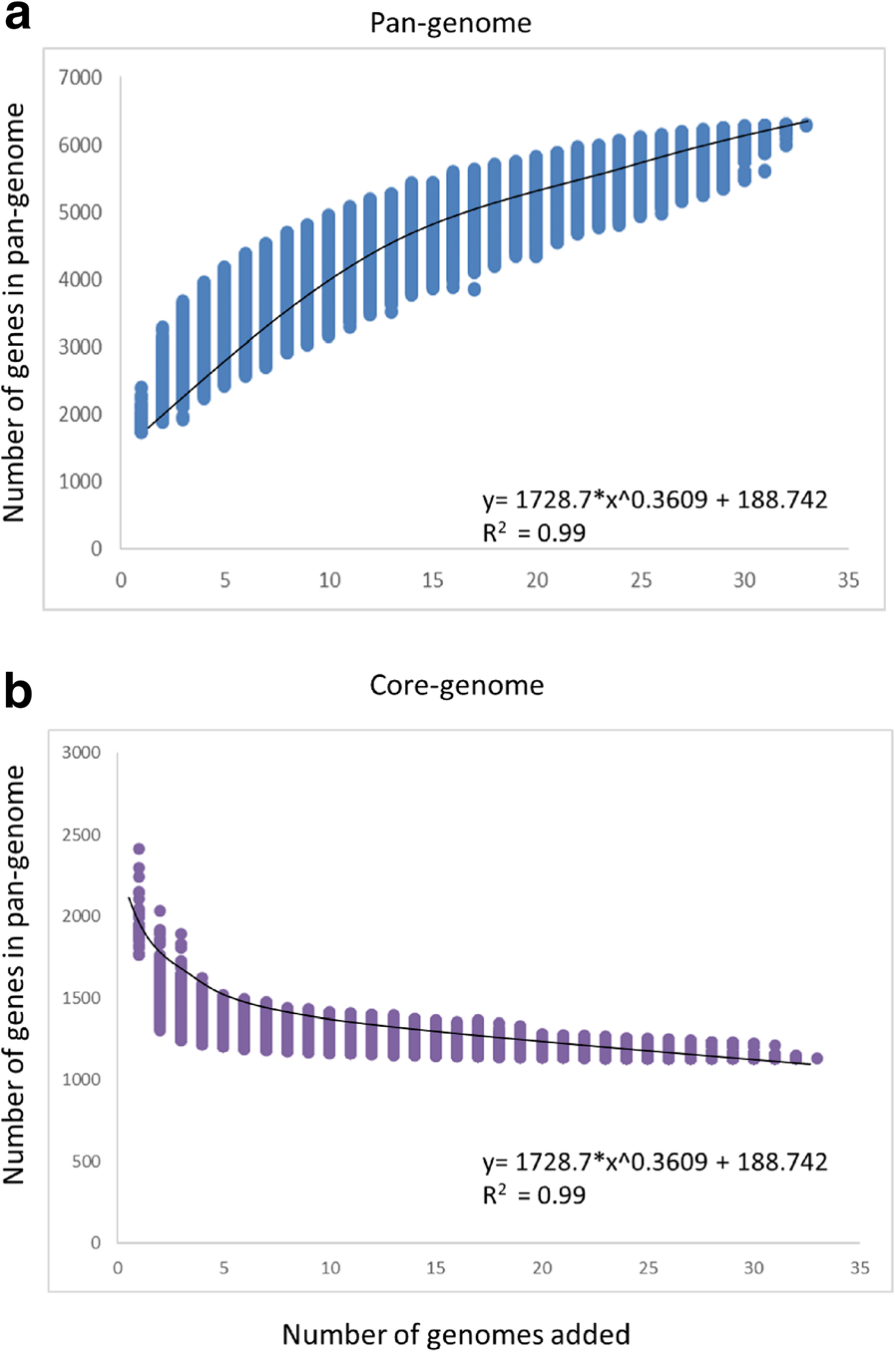
Pan-genome and core-genome of *B. longum.* **a** The pan-genome plot is of the accumulated number of new genes against the number of genomes added. **b** The core-genome plot is represented by the accumulated number of genes attributed to the core-genomes versus the number of added genomes. The resulting functions for both plots are also reported

The resulting pan-genome curve suggests an almost closed *B. longum* genome, which grows by an average of 150 genes per genome for the first thirty iterations after which the number of new gene families begins to decrease (Fig. 2, panel a). This means that, after addition of the 30^th^ genome, any further genome additions will result in only minor increases of the pan-genome. Analysis of the core-genome, which represents those gene families for which there is a member present in each of the strains analysed, reveals an asymptotic trend, essentially stabilizing after the 30^th^ genome addition at 1145 genes (Fig. 2, panel b) [38].

To identify genes that may confer a species-specific adaption, the core *B. longum* genome was compared to those of *B. breve, B. bifidum, B. animalis* and *B. adolescentis* which have been reported elsewhere [6, 7, 39]. Such differences may be a reflection of different colonisation strategies employed by the *B. longum* species. The most striking difference is the presence of *B. longum*-specific genes involved in the transport and metabolism of certain carbohydrates. More specifically, the *B. longum* core-genome contains genes that are predicted to encode xylanases, arabinofuranosidases, and associated ABC transporters which were found to be absent in the *B. breve, B. bifidum, B. animalis* and *B. adolescentis* core genomes. This indicates that the conserved ability to metabolise certain, possibly xylose/arabinose-containing plant-derived carbohydrates confers an adaptive advantage to the *B. longum* species.

### Phylogenetic analysis of *B. longum* indicates a novel subspecies

Up until very recently, three *B. longum* subspecies were recognized, i.e. *longum, infantis* and *suis*, although originally classified as three separate species [40–42]. The most recent classification of the three *B. longum* subspecies is summarised in Additional file 3: Table S4. This summary briefly outlines the environments from which each subspecies has been isolated, typical cell morphology of each subspecies and subspecies-specific carbohydrate fermentation patterns. A fourth subspecies, *B. longum* subsp. *sullium*, was recently recognized based on multi-locus and amplified–fragment length polymorphism approaches and urease activity, members of which had previously been classified as *B. longum* subsp. *suis* [43].

**Fig. 3:**
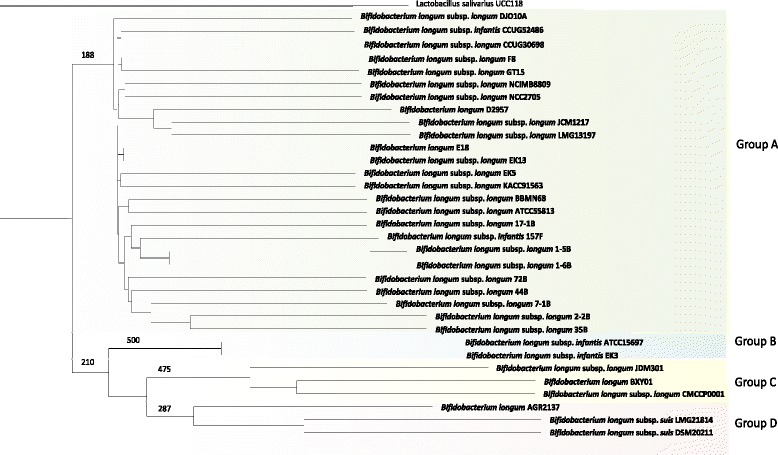
Phylogenetic analysis of *B. longum.* A phylogenetic supertree showing the relationship between thirty three complete and incomplete bifidobacterial strains and *L. salivarius* UCC118 as an outlier

In order to analyse the phylogeny of the thirty three sequenced *B. longum* strains, which include representatives of three *B. longum* subspecies, a *B. longum* phylogenetic supertree was constructed employing the deduced protein sequences of the *B. longum* core-genome as conserved molecular markers (Fig. 3). A sequenced representative of the fourth *B. longum* subspecies was not available and so could not be included in this study. As illustrated from the resulting consensus tree, two major clades are evident (Fig. 3). The first clade consists of a group of twenty five highly related strains, the majority of which are members of the subspecies *longum* taxonomic group (Fig. 3; Group A). Interestingly, *B. longum* subsp. *infantis* 157 F and *B. longum* subsp. *infantis* CCUG 52486 are placed within this cluster. It is evident from the resulting consensus tree that both of these strains have been taxonomically miss-assigned and are in fact members of the subspecies *longum.*

The second clade is split into three distinctive phylogenetic groups (Fig. 3; Groups B, C and D). As expected the three (non-human) animal isolates, *B. longum* subsp. *suis* LMG21814 (isolated from porcine faeces), *B. longum* subsp. *suis* DSM20211 (isolated from porcine faeces) and the non-subspeciated strain *B. longum* AGR2137 (isolated from calf faeces) are clustered together (Fig. 3; Group D). The phylogenetic analysis performed here did not result in the identification of the subspecies *sullium* group, which is not surprising as no representative genome of this subspecies was available.

There is no clear separation between *B. longum* subsp. *infantis* ATCC 15697 and *B. longum* subsp. *infantis* EK3, which form group B, a single subspecies *infantis* phylogenetic group (Fig. 3; Group B). Interestingly, three strains (*B. longum* subsp. *longum* JDM301, *B. longum* subsp. *longum* CMCCP0001 and *B. longum* BXY01) form a separate phylogenetic group (Fig. 3; Group C), indicative of a fourth phylogenetic subgroup. Both *B. longum* subsp. *longum* JDM301 and *B. longum* BXY01 are human isolates, whereas *B. longum* subsp. *longum* CMCCP0001 represents a faecal isolate from a healthy infant. It is also worth noting that both *B. longum* subspecies *longum* JDM301 and *B. longum* subspecies *longum* CMCCP0001 are routinely used in commercial probiotic products [44, 45]. *B. longum* subsp. *longum* JDM301 has been routinely grown in a stable and rich nutritional medium for a long period of time, whereas *B. longum* subsp. *longum* CMCCP0001 has been used in probiotic compounds for more than twenty years [44, 45]. Therefore, it is possible that this phylogenetic group has formed from strains that have evolved within industrial settings (Fig. 3; Group C). However, this cannot be established with certainty as it is unknown whether *B. longum* BXY01 is a commercially used strain.

Interestingly however, of all strains considered in this phylogenetic analysis and with the exception of *B. longum* subsp. *longum* BBMN68, the strains in Group C are the only Chinese isolates. Therefore, it is also possible that this fourth phylogenetic subgroup may represent a fifth subspecies which may be more prevalent amongst the Chinese population. However, validation of such a fifth subspecies and its possible geographical delineation will require further genome sequencing of additional *B. longum* isolates.

A phylogenetic approach using seven house-keeping genes has previously been used to differentiate between bifidobacterial (sub) species and it was found that this method allowed for a significant increase in the discriminatory power between taxa as compared to more traditional methods, for example 16 s rRNA alignments [46]. This approach was explored using several housekeeping genes and the resulting phylogenetic tree was the same as that generated from the core *B. longum* genome (Data not published).

### *In silico* prediction of *B. longum* metabolic capabilities

**Fig. 4:**
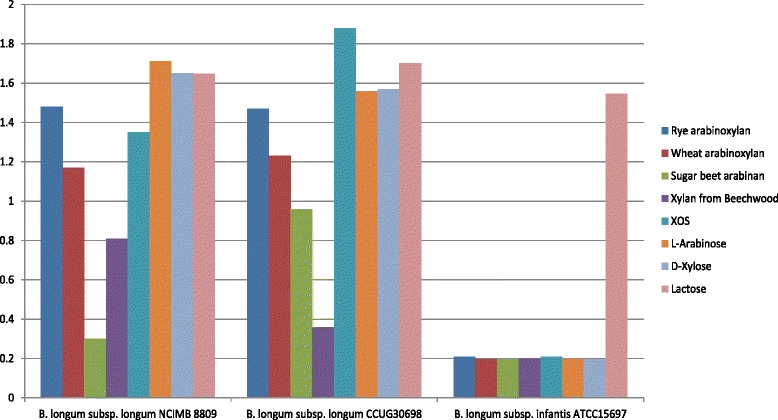
Carbohydrate metabolism by *B. longum.* Final OD600 values following 24 h growth of various *B. longum* strains grown on 1 % rye arabinoxylan, 1 % wheat arabinoxylan, 1 % sugar beet arabinan, 1 % xylan from beech wood, 1 % xylo-oligosaccharide (XOS), 1 % arabinose, 1 % xylose and 1 % lactose

To elucidate possible correlations between the genomic content of the analysed *B. longum* strains and particular phenotypic abilities, we analysed the predicted metabolic capabilities of each strain (as summarised in Table 3). Taking into account the current subspecies classification, *B. longum* subsp. *infantis* ATCC 15697 and *B. longum* subsp. *infantis* EK3 are characteristic representatives of the *infantis* subspecies (Additional file 3: Table S4). The genome sequences of both strains reveal that they are typical human milk oligosaccharide (HMO) utilisers and that they encode a considerably lower number of GHs that are associated with the catabolism of plant-derived polysaccharides (Table 3). This was verified phenotypically for *B. longum* subsp. *infantis* ATCC 15697, which is unable to utilise plant-derived polysaccharides and the pentose sugars arabinose and xylose, which represent the monosaccharides that are found in many plant-derived polysaccharides (Fig. 4).

**Table 3:** Presence or absence of LNB/GNB, sialic acid and HMO gene clusters in all *B. longum* genomes

	LNB/GNB	Sialic acid	HMO cluster	Total number of GHs	GHs active against plant polysaccharide	Arabinose/Xylose metabolism
*B. longum* subsp. *infantis* ATCC 15697	+	+	+	25	2	−
*B. longum* subsp. *infantis* EK3	+	+	+	26	2	+ (Xylose only)
*B. longum* subsp. *suis* LMG 21814	+	+ (with the exception of *nanH)*	−	32	5	+
*B. longum* subsp. *suis* DSM 20211	+	+ (with the exception of *nanH)*	−	35	8	+
*B. longum* AGR2137	+	−	−	38	7	+
*B. longum* subsp. *longum* 72B	+	−	−	37	7	+
*B. longum* subsp. *longum* EK5	+	−	−	34	8	+
*B. longum* subsp. *longum* GT15	+	−	−	36	9	+
*B. longum* subsp. *longum* JDM301	+	−	BLJ_0318 – BLJ_0322	37	9	+
*B. longum* subsp. *longum* KACC91563	+	−	−	39	9	+
*B. longum* subsp. *longum* BXY01	+	−	BXY01_0319 – BXY01_0323	37	10	+
*B. longum* subsp. *longum* CMCCP0001	+	−	CMCCP0001_1673 – CMCCP0001_1677	38	10	+
*B. longum* subsp. *infantis* 157 F	+	−	−	41	10	+
*B. longum* subsp. *longum* BBMN68	+	−	−	38	10	+
*B. longum* subsp. *longum* 171B	+	−	−	43	11	+
*B. longum* subsp. *longum* E18	+	−	−	37	11	+
*B. longum* subsp. *longum* F8	+	−	−	43	11	+
*B. longum* subsp. *longum* NCIMB 8809	+	−	−	38	13	+
*B. longum* subsp. *longum* D2957	+	−	−	39	12	+
*B. longum* subsp. *longum* EK13	+	−	−	39	12	+
*B. longum* subsp. *longum* JCM 1217	+	−	−	38	12	+
*B. longum* subsp. *longum* LMG 13197	+	−	−	37	13	+
*B. longum* subsp. *longum* NCC2705	+	−	−	33	13	+
*B. longum* subsp. *longum* 15B	+	−	−	39	15	+
*B. longum* subsp. *longum* DJO10A	+	−	−	45	16	+
*B. longum* subsp. *infantis* CCUG 52486	+	−	−	43	17	+
*B. longum* subsp. *longum* ATCC 55813	+	−	−	48	17	+
*B. longum* subsp. *longum* CCUG 30698	+	−	−	45	18	+
*B. longum* subsp. *longum* 16B	+	−	−	50	19	+
*B. longum* subsp. *longum* 22B	+	−	−	50	19	+
*B. longum* subsp. *longum* 35B	+	−	−	50	19	+
*B. longum* subsp. *longum* 44B	+	−	−	50	19	+
*B. longum* subsp. *longum* 71B	+	−	−	50	19	+

The + symbol indicates the presence of an entire cluster and the − symbol indicates the absence of an entire cluster

*In silico* analysis revealed that group A strains (Fig. 3) are characteristic members of the *longum* subspecies (Additional file 3: Table S4) and are tailored towards the metabolism of plant-derived carbohydrates (Table 3). A high variability in the number of predicted GHs, specialised in the degradation of plant polysaccharides, was observed across all analysed genomes from these Group A subspecies *longum* representatives (Table 3). Phenotypic analysis demonstrated the ability of *B. longum* subsp. *longum* NCIMB 8809 and *B. longum* subsp. *longum* CCUG 30698 to utilise various plant-derived polysaccharides and plant-associated pentose sugars (Fig. 4). As is characteristic for members of this subspecies, the strains located in group A lack the majority of genes that have previously been shown to be required for HMO utilisation (Table 3).

Both *B. longum* subsp. *suis* strains analysed adhere to the current classification of the *suis* subspecies as both strains are isolated from porcine faeces and have a predicted ability to metabolise a range of different carbohydrates including pentoses (Fig. 3, Table 3). In addition, both strains encode a near complete sialic acid metabolism cluster, yet appear to lack the sialidase-encoding *nanH*, presumed to be responsible for removing terminal sialic acid residues from oligosaccharides found in HMOs and/or mucin [47]. Though *nanH* is absent in both strains, they may still be capable of growth on sialic acid as each strain is predicted to possess the transport system necessary for the internalisation of sialic acid [47]. The third animal isolate, *B. longum* AGR2137, is more similar to members of the subspecies *longum* as this strain only harbours a partial sialic acid metabolism cluster.

Genomic data analysis of the strains *B. longum* subsp. *longum* BXY01, *B. longum* subsp. *longum* JDM301 and *B. longum* subsp. *longum* CMCCP0001 provides further evidence that these strains may represent a distinct subspecies (Fig. 3). These strains do not fit the current classification for the three *B. longum* subspecies as these strains are predicted to metabolise plant-derived polysaccharides, yet also appear to possess a considerable number of HMO-metabolising capabilities (Table 3 and Additional file 3: Table S4).

### Assessment of genetic accessibility and methylome analysis

**Fig. 5:**
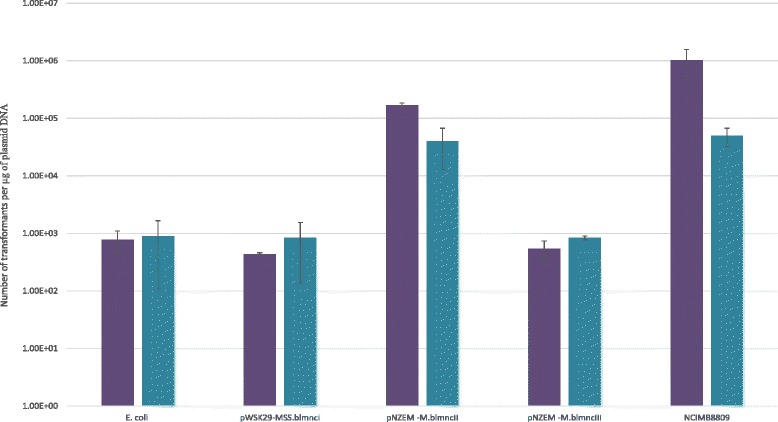
Genetic accessibility of *B. longum* subsp. *longum* NCIMB 8809. Transformation efficiency of *B.longum* subsp. *longum* NCIMB 8809 using pAM5 (purple bars) and pPKCM7 (blue bars) isolated from *E.coli* EC101*,* pWSK29-MSS.blmncI, pNZEM-M.blmncII, pNZEM-M.blmncIII and *B.longum* subsp. *longum* NCIMB 8809

Firstly, to investigate how genetically accessible *B. longum* subsp. *longum* NCIMB 8809 and *B. longum* subsp. *longum* CCUG 30698 are, the transformation frequency of two *E. coli-*bifidobacterial shuttle vectors, pPKCM7 and pAM5 was determined (Table 4). The approach for this involved introducing plasmid DNA that had originated from both *B. longum* subsp. *longum* strains (i.e. plasmid DNA that is protected from native R-M systems) whereas plasmid DNA isolated from *E. coli* EC101 was used as a control. It is worth noting that, as the plasmid DNA is isolated from two different microorganisms with different isolation protocols, there may be differences in the purity of plasmid DNA used for transformation, which could be a confounding factor. A three-log increase in transformation efficiency was observed for pAM5 and a single-log increase in transformation efficiency was observed for pPKCM7 when the respective plasmid DNA had been isolated from *B. longum* subsp. *longum* NCIMB 8809 relative to the situation when DNA isolated from *E. coli* had been used for the transformation (Fig. 5). Unfortunately, we were unuable to introduce either of the two plasmids in *B. longum* subsp. *longum* CCUG 30698 and therefore could not test this strain. These results indicate that *B. longum* subsp. *longum* NCIMB 8809 encodes functional R-M systems that negatively impact on the transformation efficiency of this strain when unmethylated DNA is used. *B. longum* subsp. *longum* CCUG 30698 is also likely to encode one or more potent R-M systems given its complete recalcitrance to transformation.

**Table 4:** Bacterial strains and plasmids used in this study

Strain/plasmid	Relevant characteristics	Additional Information	Reference or source
*E.coli* strains			
EC101	Cloning host, repA^+^ km^r^		[51]
EC101 pNZEM-M.blmncII	Containing *M.blmNCII*	Type II R-M MTase *B8809_0607*	This study
EC101 pNZEM -M.blmncIII	Containing *M.blmNCIII*	Type II R-M MTase *B8809_0958*	This study
EC101 pWSK29-MS.blmncI	Containing *MS1.blmNCI*	Type I R-M MTase and HsdS1 *B8809_1353* and *B8809_1354*	This study
EC101 pNZ44-S.blmncI	Containing *S2.blmNCI*	Type I R-M HsdS2 *B8809_1352*	This study
EC101 pWSK29-MSS.blmncI	Containing *MS1.blmNCI* and *p44 + S2.blmNCI*	Type I R-M MTase, HsdS1 and HsdS2 *B8809_1353, B8809_1354* and *B8809_1352*	This study
*B.longum* strains			
NCIMB 8809	Isolated from nursling stool		
CCUG 30698	Isolated from adult intestine		
NCIMB 8809-ArfB	pORI19-tetMod-ArfB insertion mutant of NCIMB 8809	579 bp internal fragment of *arfB* and *tetMod* gene	This study
Plasmids			
pAM5	pBC1 –puC19-Tc^r^		[70]
pPKCM7	pblueCm harbouring rep pCIBA089		[71]
pNZ44	Cm^r^, expression vector		[72]
pNZ44-S.blncI	pNZ44 derivative containing *blncIS*		This study
pWSK29			[73]
pNZ8048-Em	Emr; nisin-inducible translational fusion vector		[74]
pNZEM -M.blmncII	pNZEM derivative containing *M.blmNCII*	Type II R-M MTase *B8809_0607*	This study
pNZEM -M.blmncIII	pNZEM derivative containing *M.blmNCIII*	Type II R-M MTase *B8809_0958*	This study
pWSK29-MS.blmncI	pWSK29 derivative containing *MS1.blmNCI*	Type I R-M MTase and HsdS1 *B8809_1353* and *B8809_1354*	This study
pNZ44-S.blmncI	pNZ44 derivative containing *S2.blmNCI*	Type I R-M HsdS2 *B8809_1352*	This study
pWSK29-MSS.blmncI	pWSK29 derivative containing *MS1.blmNCI* and *p44* + *S2.blmNCI*	Type I R-M MTase, HsdS1 and HsdS2 *B8809_1353, B8809_1354* and *B8809_1352*	This study
pORI19	Emr, repA-, ori+, cloning vector		[51]
pORI19-tet-ArfB	Internal 579 bp fragment of *arfB* and tetW cloned into pORI19		This study

Genome analysis of *B. longum* subsp. *longum* NCIMB 8809 and *B. longum* subsp. *longum* CCUG 30698 clearly indicates that each strain specifies one or more complete R-M systems and/or orphan MTases (Table 5). Methylome analysis was also employed (See Materials and Methods) which allowed for the identification of a number of distinct methylation recognition sequences (Table 5). It should be noted that methylated cytosines are not faithfully detected and require specific detection methods [48, 49].

**Table 5:** R-M systems of *B. longum* subsp. *longum* NCIMB 8809 and *B. longum* subsp. *longum* CCUG 30698

Strain	R-M type	Locus tag	Predicted function	REBASE comparison	Predicted associated motif	nSites on pAM5
*B. longum* subsp. *longum* NCIMB 8809	I	*B8809_1350 (S3.blmNCI)*	HsdS subunit	100 % with Bbr12LORF1341	5'–GTNNNNNTGCC–3'/	1
		*B8809_1351*	Recombinase	-		
		*B8809_1352(S2.blmNCI)*	HsdS subunit	100 % with Bbr12LORF1345		
					3'CTNNNNNACGG–5'*	
		*B8809_1353(S1.blmNCI)*	HsdS subunit	100 % with Bbr12LORF1345		
		*B8809_1354(M.blmNCI)*	MTase	100 % with BloF8ORF1960P		
		*B8809_1355 (R.blmNCI)*	RTase	100 % with BloF8ORF1960P		
	II	*B8809_0606 (R.blmNCII)*	RTase	90 % with Blo68ORF557P	5' – CWGG – 3'	13
		*B8809_0607(M.blmNCII)*	MTase	82 % with Blo68ORF556P, N5-C		
	II	*B8809_0959 (M.blmNCIII)*	MTase	67 % with Bde27679ORF2230P, N4-C	5' – TCGGCGA – 3'*	0
*B. longum* subsp. *longum* CCUG 30698	I	*BBL306_1749*	HsdS subunit	42 % with Cty7086ORFEP	5' –ACCNNNNNRTTG– 3'/ 3' –TGGNNNNNYAAC– 5'*	2
		*BBL306_1750*	Recombinase	-		
		*BBL306_1751*	HsdS subunit	44 % with BkaPV202ORF3675		
		*BBL306_1752*	Hypothetical protein	-		
		*BBL306_1753*	MTase	98 % with Blo44BORF2305P	5'–GCNNNNNNNNTGC–3'*	4
		*BBL306_1754*	HsdS subunit	67 % with BloDORF1956P		
		*BBL306_1756*	RTase	100 % with BloDORF1959P		
	II	*BBL306_0230*	RTase	100 % with BloAORF289P	5' – CTGCG – 3'*	1
		*BBL306_0231*	MTase	68 % with BbrUIII, N6-A		
	II	*BBL306_0271*	MTase	100 % with M.Blo7210ORFAP, N6-A	Unknown	Unknown
	II	*BBL306_1693*	MTase	10 % Pae9BRORFBP,N4-C/N6-A	Unknown	Unknown
	IIG	*BBL306_1028*	RTase/MTase	98 % with Blo1217ORF992P, N6-A	5' – CGGGG – 3'*	5
	III	*BBL306_0910*	MTase	45 % with Bwa914ORF4853, N6-A	Unknown	Unknown

Methylated bases are indicated in bold red text. The “*” indicates sites that were identified by methylome analysis

This analysis revealed the recognition motif 5′-G^6m^ATNNNNNTGCC-3′ in *B. longum* subsp. *longum* NCIMB 8809, and the two recognition motifs 5′-ACCNNNNNRTTG-3′ and 5′-GCANNNNNNNNTGC-3′ in *B. longum* subsp. *longum* CCUG 30698 (Table 5). These motifs are reminiscent of type I R-M recognition sequences [16] (Table 5). Both strains are indeed predicted to encode a single and apparently complete type I R-M system encompassing a single HsdM subunit, multiple HsdS subunits, and a one HsdR subunit, responsible for modification, restriction and sequence recognition specificity (Table 5, Additional file 4: Figure S2). It has been observed that in other organisms possessing multiple HsdS subunit-encoding genes, novel specificities can be generated by recombination events among such *hsdS* sequences [50].

*B. longum* subsp. *longum* NCIMB 8809 is also predicted to encode one complete type II R-M system and one orphan type II R-M N-4 cytosine MTase (Table 5). Based on REBASE interrogation, the REase associated with the complete type II R-M system is predicted to recognize the DNA sequence, 5′-CCWGG-3′ (Table 5). However, methylome analysis only identified one type II recognition motif, 5′-TCGG^m4^CCGA-3′, which we predict to be associated with the presumed orphan type II MTase (Table 5)*. B. longum* subsp. *longum* CCUG 30698 is also predicted to encode a type II R-M system that is represented by an REse and N6-adenine MTase. Based on comparisons to REBASE and methylome analysis, we predict that this type II R-M system recognises the motif, 5′-CTGC^m6^AG-3′ (Table 5). REBASE searches also revealed the presence of what appears to be two orphaned type II MTases and an incomplete type III R-M system (Table 5). Finally, a potential type IIG R-M system was identified; based on the REBASE searches and methylome data we predict that this R-M is associated with the recognition motif 5′-CGGG^m6^AG-3′ (Table 5).

### Individual effects of *B. longum* R-M systems on transformation efficiency

Based on the methylome data obtained and comparative analysis performed on the identified R-M systems, we wanted to verify that both *B. longum* strains encode MTases that protect the respective genomic DNA. This could only be tested for those R-M systems that are predicted to recognise the motifs 5′-CCWGG-3′ and 5′-CTGCAG-3′ as commercial restriction enzymes that recognise these particular motifs are available. As expected the genomic DNA from *B. longum* subsp. *longum* NCIMB 8809 was protected from restriction with EcoRII (which cuts 5′-CCWGG-3′ sequences), while it was digested by PstI (which targets 5′-CTGCAG-3′ sequences). Conversely and as expected, genomic DNA from *B. longum* subsp. *longum* CCUG 30698 was protected from restriction with PstI, while it was cut by EcoRII (Additional file 5: Figure S3).

The type II R-M systems of *B. longum* subsp. *longum* NCIMB 8809 encompass the MTase-encoding genes *blmNCII* and *blmNCIII*, while the type I R-M system encompasses the HsdM-encoding gene *M.blmNCI*, and the (partial) HsdS-encoding genes *S1.blmNCI, S2.blmNCI* and *S3.blmNCI.* The effect of each individual R-M system on the transformation efficiency of *B. longum* subsp. *longum* NCIMB 8809 was investigated, whereas we did not pursue this for *B. longum* subsp. *longum* CCUG 30698 as this strain was non-transformable (see above). For this purpose, each gene encoding an MTase and specificity subunit (in the case of the type I system) were cloned in *E. coli* EC101 and with the exception of *S2.blmNCI*, all genes were cloned together with their presumed native promoter-containing region (see Materials and Methods).

Plasmids pAM5 and pPKCM7 were introduced into *E. coli* EC101 derivatives that harboured plasmids pWSK29-MSS.blmncI or pNZEM-M.blmncII. Plasmid preparations of the resulting pAM5 and pPKCM7-containing *E. coli* EC101-derivative strains were used for *B. longum* subsp. *longum* NCIMB 8809 transformation. As positive controls pAM5 and pPKCM7 plasmid DNA was isolated from *B. longum* subsp. *longum* NCIMB 8809 (i.e. pAM5 and pPKCM7 methylated by and thus protected against from the native R-M systems), whereas pAM5 and pPKCM7 plasmid DNA isolated from *E. coli* EC101 acted as the negative control (i.e. unprotected pAM5 and pPKCM7).

The highest observed transformation efficiency was achieved when plasmid DNA was isolated from the *E. coli* EC101 derivative harbouring plasmid pNZEM-M.blmncII (Fig. 5). This observation is not surprising as the motif associated with this MTase has the highest number of occurrences on pAM5 compared to the other recognition motifs identified (Table 5). Our data indicate that the type I R-M system present in *B. longum* subsp. *longum* NCIMB 8809 does not have a negative effect on transformation efficiency. This result may be due to the low number of recognition motifs present on pAM5 that are associated with this type I R-M system or to the possibility that this system is inactive (Table 5). The transformation efficiency for the positive control (pAM5 plasmid DNA isolated from *B. longum* subsp. *longum* NCIMB 8809) is still 10-fold higher than that achieved for plasmid DNA isolated from the *E. coli* EC101 derivative harbouring plasmid pNZEM-M.blmncII, which suggests that the latter plasmid is not fully protected against the endogenous restriction activity of NCIMB 8809. Furthermore, the second type II R-M system present in *B. longum* subsp. *longum* NCIMB 8809 does not negatively impact on the transformation efficiency of this strain and may be due to the fact that this R-M system is non-functional.

### Disruption of the *arfB* in *B. longum* subsp. *longum* NCIMB 8809

The *arfB* gene was selected as a mutational target in order to determine whether methylation of the non-replicating plasmid by the *B. longum* subsp. *longum* NCIMB 8809 MTase M.blmncII would increase transformation efficiency to allow for site-specific homologous recombination. The *arfB* gene encodes a putative α-L-arabinofuranosidase (Ara*f*) predicted to be involved in the degradation of the plant-derived carbohydrate arabinoxylan through the hydrolysis of α-L-arabinofuranosyl residues from the xylan backbone of arabinoxylan. The DNA fragment of 579 bp, representing an internal fragment of the gene *arfB, was* cloned in pORI19 to generate construct pORI19-ArfB (see Materials and Methods). The pORI19 derivative, pORI19-ArfB, was provided with the tetracycline marker, *tetW*_*Mod*_, resulting in the plasmid, pORI19-tetMod-ArfB. We used a synthetic *tetW*_*Mod*_ gene, which is free of EcoRII restriction sites compared to the original *tetW* sequence which contains a total of seven EcoRII sites (we noticed that plasmid DNA isolated from *E. coli* pNZEM-M.blmncII was not fully methylated and therefore was still vulnerable to (partial) restriction (Additional file 6: Figure S4, panel a)). There are two EcoRII sites present in the selected internal *arfB* fragment, whereas the non-replicative pORI19 plasmid contains just a single EcoRII site (and therefore pORI19-tetMod-ArfB contains three EcoRII sites). This pORI19 derivative is unable to replicate in *B. longum* subsp. *longum* NCIMB 8809 as it lacks a functional replication protein [51]. In order to methylate the constructed plasmid pORI19-tetMod-ArfB, this plasmid was introduced into *E. coli* pNZEM-M.blmncII, expressing the type II MTase from *B. longum* subsp. *longum* NCIMB 8809. Restriction analysis revealed that plasmid pORI19-tetMod-ArfB (when isolated from *E. coli* pNZEM-M.blmncII) was protected from restriction with EcoRII (Additional file 6: Figure S4, panel b). Restriction analysis also revealed that the un-methylated pORI19-tetMod-ArfB plasmid (i.e. not introduced into *E. coli* pNZEM-M.blmncII) was subject to restriction by EcoRII (data not shown).

**Fig. 6:**
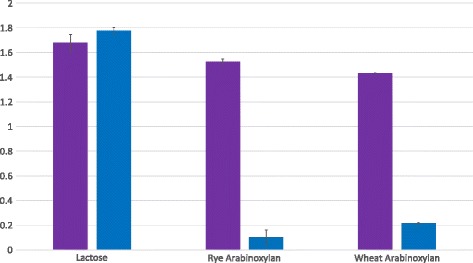
Phenotypic analysis of the *B. longum* subsp. *longum* NCIMB 8809 insertion mutant strain. Growth profile analysis of *B.longum* subsp. *longum* NCIMB 8809 (purple) and *B.longum* subsp. *longum* NCIMB 8809-ArfB (blue) in modified Rogosa broth supplemented with lactose, rye arabinoxylan or wheat arabinoxylan

Methylated pORI19-tetMod-ArfB was then introduced into *B. longum* subsp. *longum* NCIMB 8809 via electroporation. Several Tet^r^ transformants were obtained and analysed by colony PCR, which indeed verified that the individual pORI19-derivative had integrated at the expected chromosomal location. To assess the phenotypic effect of the gene disruption created in *arfB,* a selected mutant, designated *B. longum* subsp. *longum* NCIMB 8809-ArfB, was analysed for its ability to grow on mMRS supplemented with arabinoxylan as the sole carbon source. In contrast to (wild type) *B. longum* subsp. *longum* NCIMB 8809, the *arfB*-mutant exhibited a complete inability to grow on both rye and wheat arabinoxylan (though retained, as expected, the ability to grow on lactose as a sole carbon source) (Fig. 6).

This result shows that *arfB* is required for growth of *B. longum* subsp. *longum* NCIMB 8809 on arabinoxylan. Based on the predicted function of ArfB we speculate that this enzyme cleaves the arabinose substitutions from arabinoxylan and that the released arabinose moieties are then internalized and metabolised.

## Conclusions

Comparative genomics of bifidobacteria may be very helpful in order to expand our understanding of (bifido)bacterial-host interactions, whereby they may elicit health benefits, and of the ability for bifidobacteria to adapt and compete in the intestinal environment. The full genome sequencing of two human *B. longum* isolates and the subsequent analysis of all complete and incomplete *B. longum* genomes allowed for the description of the pan- and core-genome for the *B. longum* species. This analysis revealed that the *B. longum* pan-genome is essentially closed and that the genomes included in this analysis are likely to be sufficient in describing the genetics and derived biology of this bifidobacterial species.

It is evident from the determined genome sequences, which are comparable to other fully sequenced *B. longum* genomes, that *B. longum* subsp. *longum* NCIMB 8809 and *B. longum* subsp. *longum* CCUG 30698 possess various genetic adaptations and associated ecological fitness to suit life in the GIT. In light of the current *B. longum* subspecies classification, the phenotypic, phylogenetic and *in silico* analysis presented here has provided evidence for the existence of a novel phylogenetic type, which may represent a fifth subspecies. To determine whether further subclassification is desirable and can be justified, additional sequencing of *B. longum* isolates genomes is required. We also propose an amendment to the description of the subspecies *infantis* in that members may have capabilities to utilise carbohydrates other than HMOs. We also provide evidence that indicates that *B. longum* subsp. *infantis* 157 F and *B. longum* subsp. *infantis* CCUG 52486 should be assigned to the *longum* subspecies [52]. Finally, comparative analysis of *B. longum* highlights the versatility of this species and its ability to move with us from infancy to adulthood.

The genetic inaccessibility of bifidobacteria hinders the exploration of the molecular mechanisms that are responsible for its acclaimed probiotic activities. Improvement of transformation efficiency has previously been achieved by overcoming R-M systems [14, 15]. The negative impact of R-M systems on transformation efficiency of bifidobacteria has been demonstrated in *B. breve* UCC2003 [15], *B. adolescentis* ATCC 15703 [20] and *B. bifidum* S17 [53]. However, such an R-M barrier can be overcome by the appropriate pre-methylation of plasmid DNA [15, 20, 21, 54].

By exploiting our knowledge of the active R-M systems harboured by *B. longum* subsp. *longum* NCIMB 8809, we successfully generated a mutant of this strain via homologous recombination. The mutant strain *B. longum* subsp. *longum* NCIMB 8809-ArfB revealed that *arfB* plays a vital role in arabinoxylan degradation.

## Methods

### Bacterial strains and growth conditions

Bacterial strains and plasmids used in this study are detailed in Table 4. Bifidobacteria were routinely cultured in modified de Man, Rogosa and Sharpe (mMRS). This medium was made from first principles [55], though excluding a carbohydrate source, and then supplemented with 1 % (wt/vol) lactose (unless otherwise specified) and 0.05 % (wt/vol) cysteine-HCl. Cultures were incubated at 37 °C under anaerobic conditions which were maintained using an Anaerocult oxygen depletion system (Merck, Darmstadt, Germany) in an anaerobic chamber with an atmosphere of 5 % CO_2_ – 5 % H_2_ – 90 % N_2_.

*Escherichia coli* strains were cultured in LB broth (LB) [56, 57] at 37 °C with agitation. Where appropriate, growth media contained chloramphenicol (Cm; 10 μg ml^−1^ for *E. coli* and 2 μg ml^−1^ for *B. longum*), erythromycin (Em; 100 μg ml^−1^ for *E. coli*) ampicillin (Amp; 100 μg ml^−1^ for *E. coli*), tetracycline (Tet; 10 μg ml^−1^ for *E. coli* and *B. longum)* or kanamycin (Km; 50 μg ml^−1^ for *E. coli*).

### PacBio SMRT Sequencing, data assembly and methylome analysis

Chromosomal DNA from *B. longum* subsp. *longum* NCIMB 8809 and *B. longum* subsp. *longum* CCUG 30698 was isolated as previously described [58] and purified using the PowerClean DNA Clean-Up Kit by MoBio Laboratories (Carlsbad, CA). SMRT bell library preparation was performed as previously described [59, 60]. SMRT sequencing was performed on a PacBio *RS* instrument (executed by GATC Biotech, Germany) and assembled using the Pacific Biosciences SMRTPortal analysis platform (version 2.1.1). Illumina sequencing was performed by the commercial sequencing service providers Macrogen (Seoul, Republic of Korea) (using a paired-end library). The Illumina sequences obtained were then assembled with the SMRTPortal output using MIRA v3.9 (http://www.chevreux.org/projects_mira.html). Remaining gaps and quality issues were resolved using Sanger sequencing of PCR products.

To identify methylated positions the Pacific Biosciences SMRTPortal analysis platform (version 1.4) was adopted, this employs an *in silico* kinetic reference and a *t*-test based kinetic score detection of modified base positions.

### General features prediction

Prediction of putative open reading frames (ORFs) was performed using PRODIGAL prediction software (http://prodigal.ornl.gov/) and supported by BLASTX [61] alignments. Results of Prodigal/BLASTX were combined manually and a preliminary identification of ORFs was performed on the basis of BLASTP [61] analysis against a non-redundant protein database provided by the National Centre for Biotechnology (http://www.ncbi.nlm.nih.gov/). Using the ORF finding outputs and associated BLASTP results, Artemis [62] was employed for visualisation and manual editing in order to verify, and, where necessary, redefine the start of every predicted coding region, or to remove or add coding regions. The assignment of protein function to predicted coding regions was performed manually. In addition, the individual members of the revised gene/protein data set were searched against the protein family (Pfam) [63] and COG [64] databases. Ribosomal RNA (rRNA) and transfer RNA (tRNA) genes were detected using RNAMMER (http://www.cbs.dtu.dk/services/RNAmmer/) and tRNA-scanSE (http://lowelab.ucsc.edu/tRNAscan-SE/), respectively. COG category assignment [64] was performed by means of BLASTP [61] analysis against the COG database [65] for deduced proteins of all identified ORFs contained by the genomes of both *B. longum* strains that were sequenced as part of the current study, and of all publicly available *B. longum* strains.

The genome sequences of both *B. longum* subs. *longum* strains were searched for the presence of Restriction/Modification systems using a BLASTP [61] alignment function of the REBASE database (http://rebase.neb.com/rebase/rebase.html) (cut-off e-value of 0.00001; and showing at least 30 % similarity across 80 % of the protein length).

### Pan-genome determination

Where relevant, genomes were re-annotated using the same annotation method described for *B. longum* subsp. *longum* NCIMB 8809 and *B. longum* subsp. *longum* CCUG 30698. For the available bifidobacterial genomes (complete and incomplete) a pan-genome computation was performed using PGAP v1.2 [66], which performs this analysis according to Heaps law pan-genome model [38]; the ORF content of each genome is organised in functional gene clusters using the GF (Gene Family) method and a pan-genome profile was then built.

### Phylogenetic analysis and Whole genome alignments

The computation of a phylogenetic supertree was performed based on the alignment of a set of orthologous proteins defined by the pan-genome computation. Each protein family was aligned using CLUSTAL_W v1.83 [67]. Phylogenetic trees were computed using the maximum-likelihood in PhyML v3.0 [68] and concatenated; the resulting consensus tree was computed using the Consense module from the Phylip package v3.69 using the majority rule method (http://evolution.genetics.washington.edu/phylip.html). Whole genome comparisons of the two newly sequenced *B. longum* strains were performed against *B. longum* subsp. *longum* NCC2705 (AE014295). Whole genomes were compared at the nucleotide level using MUMmer software [69] at default settings.

### Nucleotide sequence accession numbers

All sequences used for our analysis were retrieved from the GenBank database and are listed with associated accession numbers in Additional file 7: Table S1.

### DNA manipulations

Chromosomal DNA was isolated from *B. longum* subsp. *longum* NCIMB 8809 and *B. longum* subsp. *longum* CCUG 30698 as previously described [58]. Isolation of plasmid DNA from *E. coli* or *B. longum* was achieved by using the Roche High Pure Plasmid Isolation Kit (Roche Diagnostics). For *B. longum* an initial lysis step was incorporated into the plasmid isolation procedure, and cells were resuspended in lysis buffer supplemented with lysozyme (30 mg ml^−1^) and incubated at 37 °C for 30 min. The procedures for DNA manipulations were performed essentially as described by Sambrook *et al.* [56]. Restriction endonucleases, shrimp alkaline phosphatase, and T4 DNA ligase were obtained from Roche Diagnostics and used according to the supplier’s instructions (Roche Diagnostics, Bell Lane, East Sussex, United Kingdom). The synthetic single-stranded oligonucleotide primers used in this study, detailed in Additional file 8: Table S2, were synthesized by MWG Biotech AG (Ebersberg, Germany). Standard PCRs were performed using Taq PCR mastermix (Qiagen), while high-fidelity PCR was achieved by the use of either PfuUltra II polymerase (Agilent Technology) or LongAmp Taq polymerase (New England Biolabs). PCR amplicons were purified using the Roche High Pure PCR Purification Kit (Roche Diagnostics). Electroporation of plasmid DNA into *E. coli* was performed as previously described [56]. The correct orientation and integrity of all constructs was verified by DNA-sequencing, performed by Eurofins MWG Biotech.

### Construction of *B. longum* insertion mutant strains

An internal fragment of *arfB* (corresponding to locus tag B8809_1600), encompassing 579 bp and representing codons 240 through to 433 out of the 842 codons of this gene was amplified by PCR using *B. longum* subsp. *longum* NCIMB 8809 chromosomal DNA as template and the oligonucleotide primer combination arfBMfHindIII and arfBMrXbaI (Additional file 8: Table S2). The generated PCR product was ligated to pORI19, an Ori + RepA- integration plasmid [51], using HindIII and XbaI restriction sites that were incorporated into the primers for this partial *arfB*-encompassing amplicon, and introduced into *E. coli* EC101 by electroporation. Recombinant *E. coli* EC101 derivatives containing pORI19 constructs were selected on LB agar containing Em, and supplemented with X-gal (5-bromo-4-chloro-3-indolyl-D-galactopyranoside) (40 μg ml^−1^) and 1 mM IPTG.

A modified *tetW* gene, conferring resistance to tetracyclin and free of EcoRII sites, was synthesized by Eurofins Genomics, designated here as *tetW*_*Mod*_ [70]. The expected genetic structure of the recombinant plasmids pORI19-arfB (pORI19 containing an internal 579 bp fragment of the *arfB* gene) was confirmed by restriction mapping prior to subcloning of *tetW*_*Mod*_ as a SacI fragment into the unique SacI site present on pORI19-arfB. The orientation of the tetracycline resistance gene in each of the resulting plasmids pORI19-tetMod-arfB, was determined by restriction analysis. Plasmid pORI19-tetMod-arfB was introduced into *E. coli* EC101 pNZEM-M.blncbII (see below, Table 4) (transformants were selected based on Tet resistance) in order to methylate the plasmid constructs before introduction into *B. longum* subsp. *longum* NCIMB 8809. A plasmid preparation of methylated pORI19-tetMod-arfB was introduced into *B. longum* subsp. *longum* NCIMB 8809 by electroporation with subsequent selection on RCA plates supplemented with tetracycline [54].

Insertion mutants resulting from site-specific homologous recombination were initially confirmed by colony PCR targeting the tetracycline resistance genes *tetW*_*Mod*_, followed by a second confirmatory PCR adopting *tetW*_*Mod*_-based primer, either forward or reverse depending on the orientation of *tetW*_*Mod*_, in combination with a primer specific for the targeted *arfB* gene to confirm integration at the expected chromosomal position (Additional file 8: Table S2). The verified mutant carrying a chromosomal insertion in *arfB* was designated as *B. longum* subsp. *longum* NCIMB 8809-ArfB.

### Transformation of *B. longum* subsp. *longum* NCIMB 8809

Transformation of *B. longum* was achieved essentially according to a previously published protocol [54]. This protocol was optimised for *B. longum* subsp. *longum* NCIMB 8809 in order to achieve a transformation efficiency high enough to generate mutants via homologous recombination. The original protocol instructs that cultures are grown in MRS broth, a glucose-based medium. For the optimised protocol *B. longum* subsp. *longum* NCIMB 8809 was inoculated in MRS medium supplemented with 1 % lactose. Furthermore, the highest transformation efficiency for *B. longum* subsp. *longum* NCIMB 8809 was achieved when cells were harvested once they reached an OD_600_ of 0.6-0.8 (as opposed to an OD_600_ of 0.3 to 0.4 prescribed for the original protocol). For each transformation experiment a plasmid quantity of 400 ng was used (isolated from either the relevant *B. longum* strain or *E. coli* EC101) employing electroporation of *B. longum* subsp. *longum* NCIMB 8809. Transformants were selected on RCA supplemented with either chloramphenicol (Cm) for plasmid pPKCM7 or tetracycline (Tet) in the case of pAM5, and enumerated following a 48 h incubation at 37 °C under anaerobic conditions.

### Cloning of methylase-encoding genes in *E. coli*

DNA fragments encompassing the genes *M.blmNCII* (corresponding to locus tag B8809_0607), *M.blmNCIII* (corresponding to locus tag B8809_0959), *M.blmNCI* and *S1.blmNCI* (corresponding to locus tags B8809_1354 and B8809_1353, respectively), and *S2.blmNCI* (corresponding to locus tag B8809_1352) were generated by PCR amplification from chromosomal DNA of *B. longum* subsp. *longum* NCIMB 8809 using PfuUltra II polymerase and the primer combinations found in Additional file 8: Table S2. The generated PCR amplicons were digested with restriction enzymes that correspond to the sites incorporated into the 5′ end of forward and reverse primers (Additional file 8: Table S2).

The digested PCR amplicons *M.blmNCII* and *M.blmNCIII* were ligated into similarly digested pNZ8048-Em. The digested PCR amplicons *M.blmNCI* and *S1.blmNCI* were ligated into similarly digested pWSK29, whereas the digested fragment encompassing *S2.blmNCI* was ligated into similarly digested pNZ44. The resulting ligations were introduced into *E. coli* EC101 (Table 4) by electrotransformation, and transformants were selected based on kanamycin and either ampicillin (pWSK29-based), erythromycin (pNZ8048-Em-based) or chloramphenicol resistance (pNZ44-based). The plasmid content of twenty transformants was screened by restriction analysis and on average eight positive clones were identified. The integrity of five of the positively identified clones was verified by sequencing. The plasmids were designated as pNZEM-M.blmncII, pNZEM-M.blmncIII, pWSK29-MS.blmncI and pNZ44-S.blmncI (see Table 4 for details). Finally, to construct plasmid pWSK29-MSS.blmncI the DNA fragment encompassing the p44 promoter sequence and *S2.blmNCI* was generated by PCR amplification from the plasmid pNZ44-S.blmncI using LongAmp Taq polymerase and primer combination found in Additional file 8: Table S2. The digested PCR amplicon was ligated to similarly digested pWSK29-MS.blmncI and introduced into *E. coli* EC101 (Table 4) by electrotransformation, and transformants were selected based on kanamycin and ampicillin resistance. The plasmid content of twenty Kan^r^ and Amp^r^ transformants was assessed by restriction analysis resulting in the identification of sixteen positive clones. The integrity of five of the positively identified clones was verified by sequencing.

## Additional files

Additional file 1: Figure S1. Whole genome alignments. Dotplot comparison based on the genomics sequence alignments of *B. longum* subsp. *longum* NCC2705 to *B. longum* subsp. *longum* NCIMB 8809 and *B. longum* subsp. *longum* CCUG 30698. (PDF 240 kb) Additional file 2: Table S3.
*B. longum* subsp. *longum* NCIMB 8809 episome. An .xls document listing the predicted protein function of episomal ORFs located within the genome sequence of *B. longum* subsp. *longum* NCIMB 8809. (XLSX 9 kb) Additional file 3: Table S4. Summary of the *B. longum* classification. An .xls document describing the classification of the three *B. longum* subspecies, *B. longum* subsp. *longum, B. longum* subsp. *infantis* and *B. longum* subsp. *suis. (XLSX 12 kb)*
Additional file 4: Figure S2. Type I R-M systems of *B. longum.* A schematic representing the type I R-M system present in *B.longum* subsp. *longum* NCIMB 8809, *B.breve* 12 L, *B.longum* subsp. *longum* JCM1217, *B.longum* subsp. *longum* F8 and *B.longum* subsp. *longum* CCUG 30698. Each arrow represents an ORF. The predicted protein function is indicated as Restriction (Green), Modification (Red), Specificity (Blue), Recombinase (Grey), Hypothetical (White) and Transcriptional regulator (Orange). Target recognition domains (TRDs) are indicated as orange boxes. The percentage amino acid identity is indicated as compared to the *B.longum* subsp. *longum* NCIMB 8809 type I R-M. (PDF 11 kb) Additional file 5: Figure S3. Restriction analysis of *B. longum* genomic DNA. Restriction analysis of genomic DNA from *B. longum* subsp. *longum* NCIMB 8809 and *B. longum* subsp. *longum* CCUG 30698. Lane 1, molecular weight marker (Bioline). Lane 2: Unrestricted total DNA from *B. longum* subsp. *longum* NCIMB 8809, Lane 3 and 4 total *B. longum* subsp. *longum* NCIMB 8809 DNA restricted with lane 3 EcoRII and lane 4 PstI. Lane 5: Unrestricted total DNA from *B. longum* subsp. *longum* CCUG 30698, Lane 6 and 7 total *B. longum* subsp. *longum* CCUG 30698 DNA restricted with lane 6 EcoRII and lane 7 PstI. (PDF 93 kb) Additional file 6: Figure S4. Restriction analysis of plasmid DNA. a) Lane 1: molecular weight marker (Bioline). Lane 2 Unrestricted pAM5 plasmid DNA isolated from *E. coli* pNZEM-M.blmncII. Lane 3 pAM5 plasmid DNA isolated from *E. coli* pNZEM-M.blmncII and restricted with EcoRII. b) Lane 1: molecular weight marker (Bioline). Lane 2 unrestricted plasmid DNA pORI19-tetMod-ArfB isolated from *E. coli* pNZEM-M.blmncII. Lane 3 pORI19-tetMod-ArfB plasmid DNA isolated from *E. coli* pNZEM-M.blmncII and restricted with EcoRII. (PDF 102 kb) Additional file 7: Table S1. List of *Bifidobacterium longum* representatives. An .xls document with a list of all *B. longum* representatives that were used in the comparative analysis. (XLSX 13 kb) Additional file 8: Table S2. Primers used in this study. An .xls document with a list of primer used in this study and sequences of restriction sites are indicated in bold text. (XLSX 11 kb)

## Abbreviations

GIT: gastrointestinal tract
R-M system: restriction modification system
ORF: open reading frame
GF: gene family
COG: cluster of orthologous groups
PEP-PTS: phosphoenolpyruvate-phosphotransferase system
GH: glycosyl hydrolase
sEPS: surface exopolysaccharide
GT: glycosyl transferase
HMO: human milk oligosaccharide
Ara*f*: arabinofuranosidase
