# Supplementary figures and images for: Pangenome analysis of Bifidobacterium longum and site-directed mutagenesis through by-pass of restriction-modification systems
Source: BMC Genomics. 2015 Oct 21;16:832. doi: 10.1186/s12864-015-1968-4 (PMC4618763; doi:10.1186/s12864-015-1968-4)

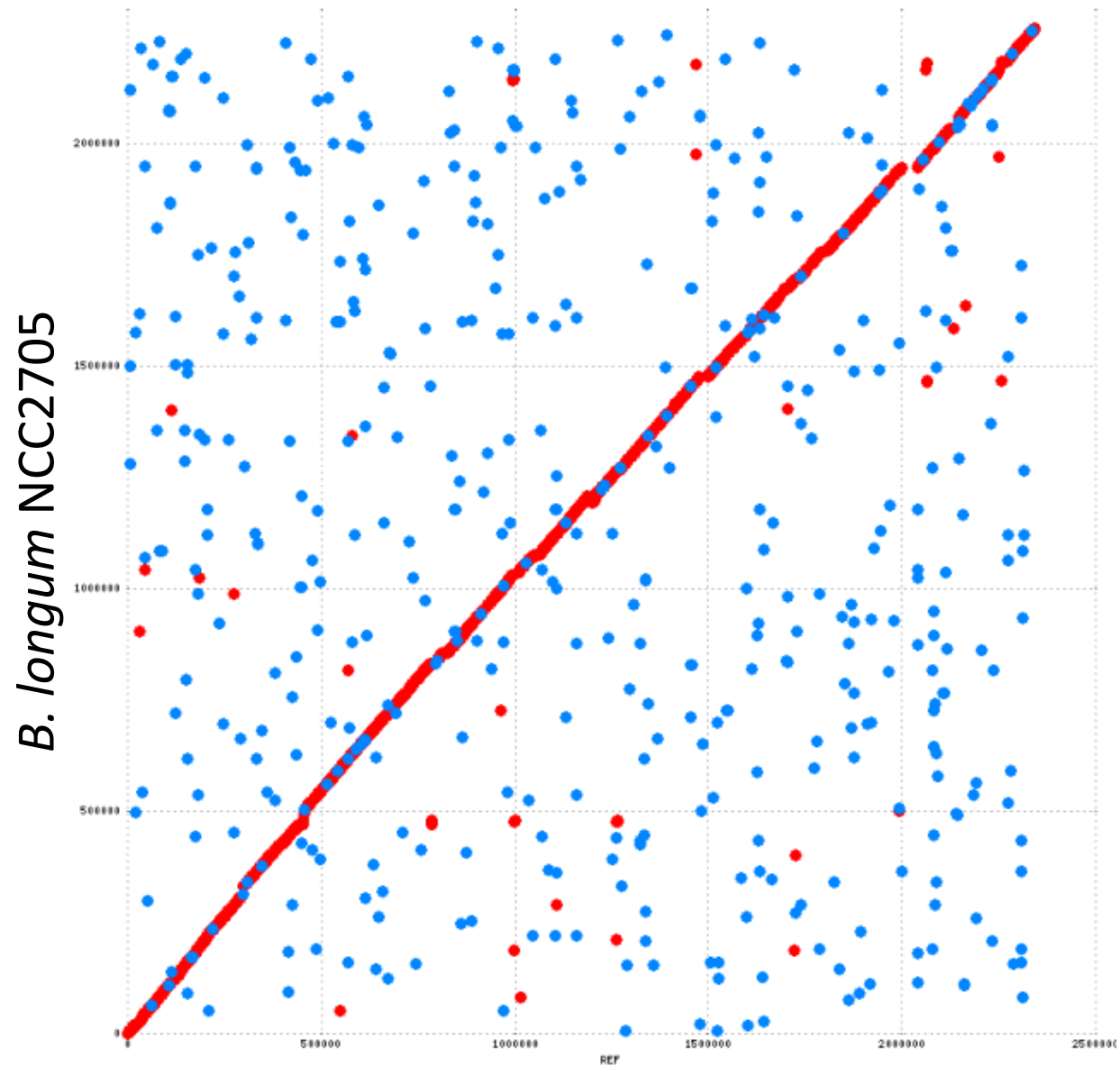

*B. longum* NCIMB8809

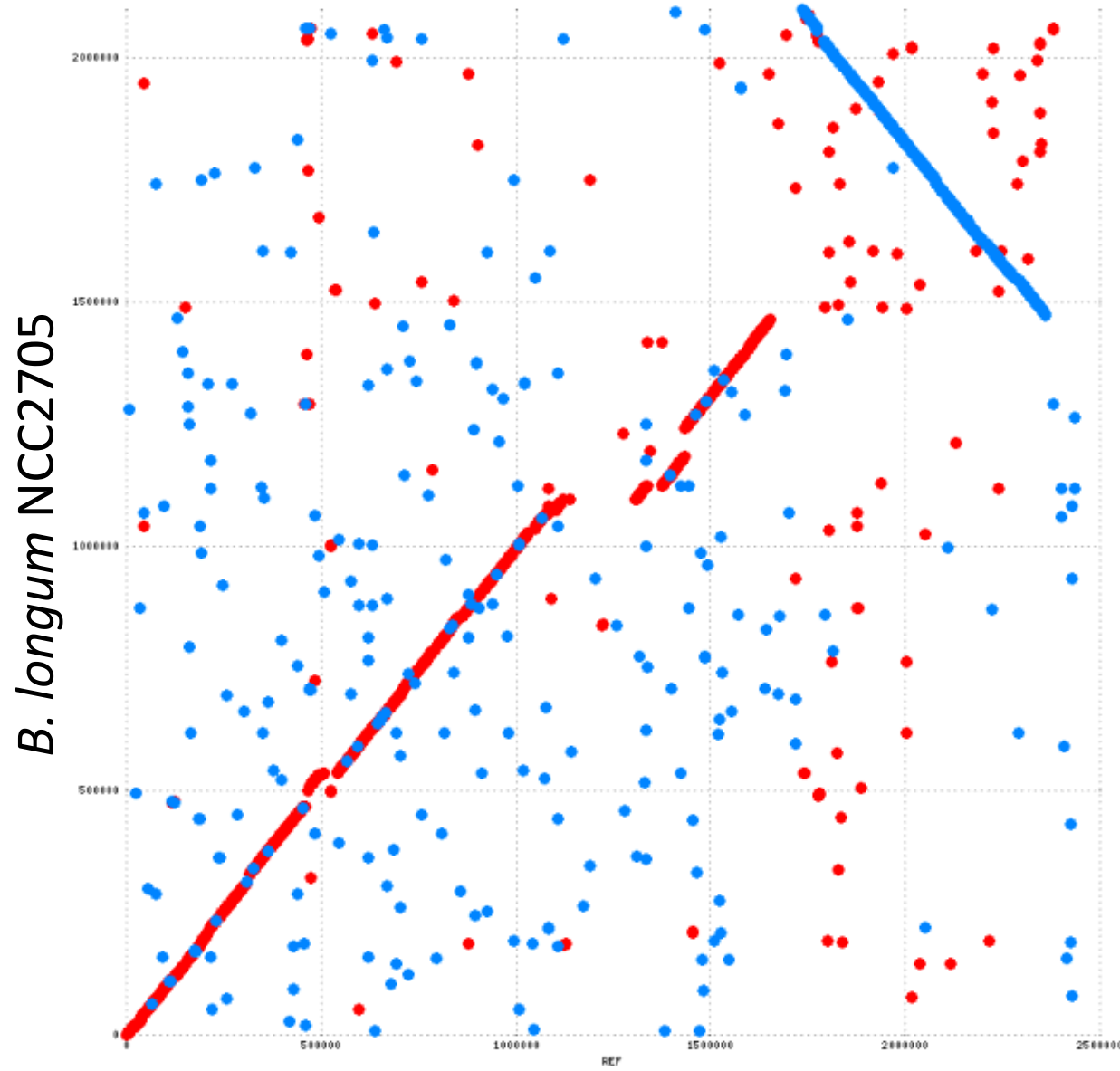

*B. longum* CCUG30698

Supplement: Additional file 1: Figure S1. — Whole genome alignments. Dotplot comparison based on the genomics sequence alignments of B. longum subsp. longum NCC2705 to B. longum subsp. longum NCIMB 8809 and B. longum subsp. longum CCUG 30698. (PDF 240 kb) [file 12864_2015_1968_MOESM1_ESM.pdf]

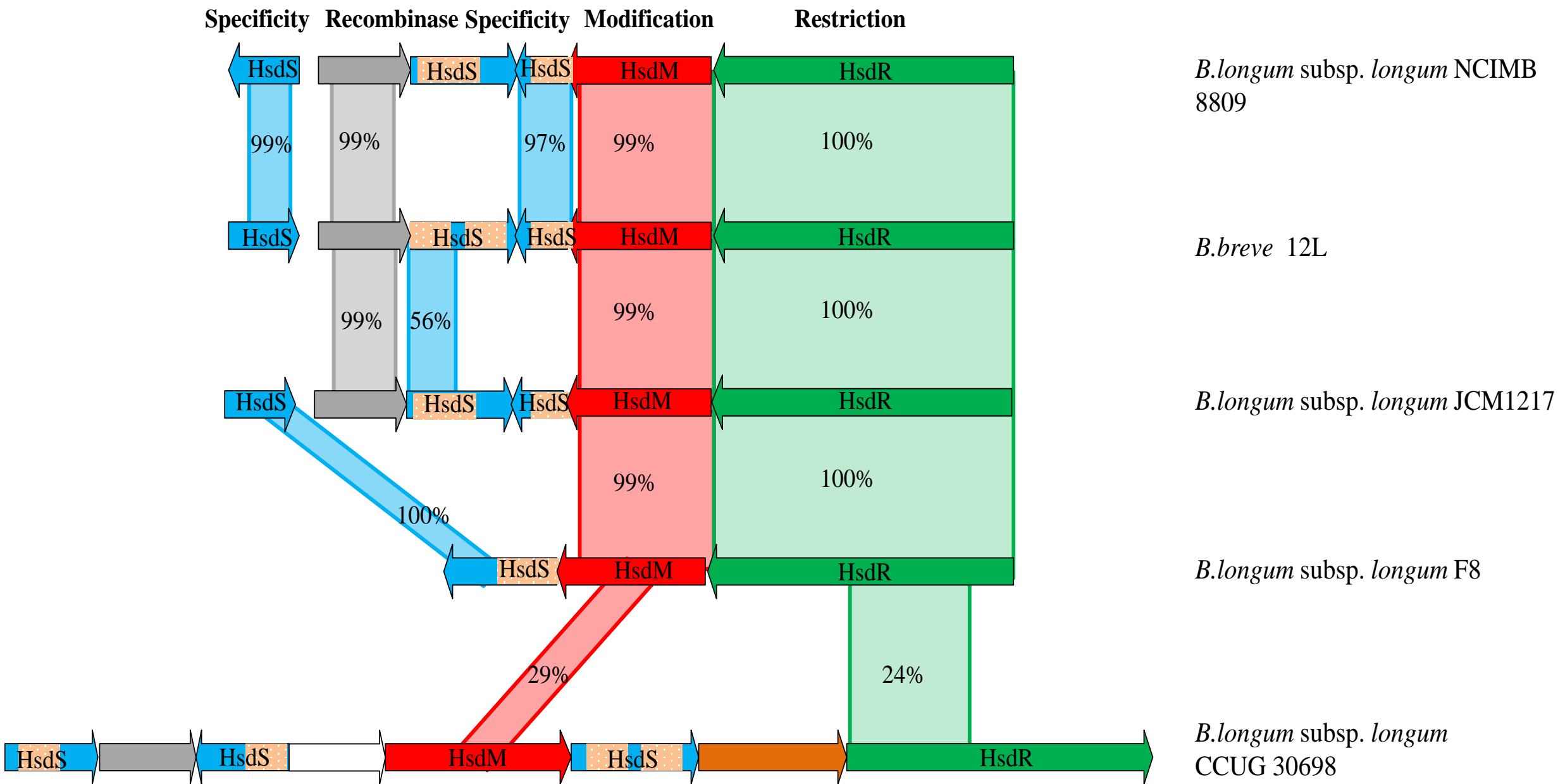

Supplement: Additional file 4: Figure S2. — Type I R-M systems of B. longum. A schematic representing the type I R-M system present in B.longum subsp. longum NCIMB 8809, B.breve 12 L, B.longum subsp. longum JCM1217, B.longum subsp. longum F8 and B.longum subsp. longum CCUG 30698. Each arrow represents an ORF. The predicted protein function is indicated as Restriction (Green), Modification (Red), Specificity (Blue), Recombinase (Grey), Hypothetical (White) and Transcriptional regulator (Orange). Target recognition domains (TRDs) are indicated as orange boxes. The percentage amino acid identity is indicated as compared to the B.longum subsp. longum NCIMB 8809 type I R-M. (PDF 11 kb) [file 12864_2015_1968_MOESM4_ESM.pdf]

1

2

3

4

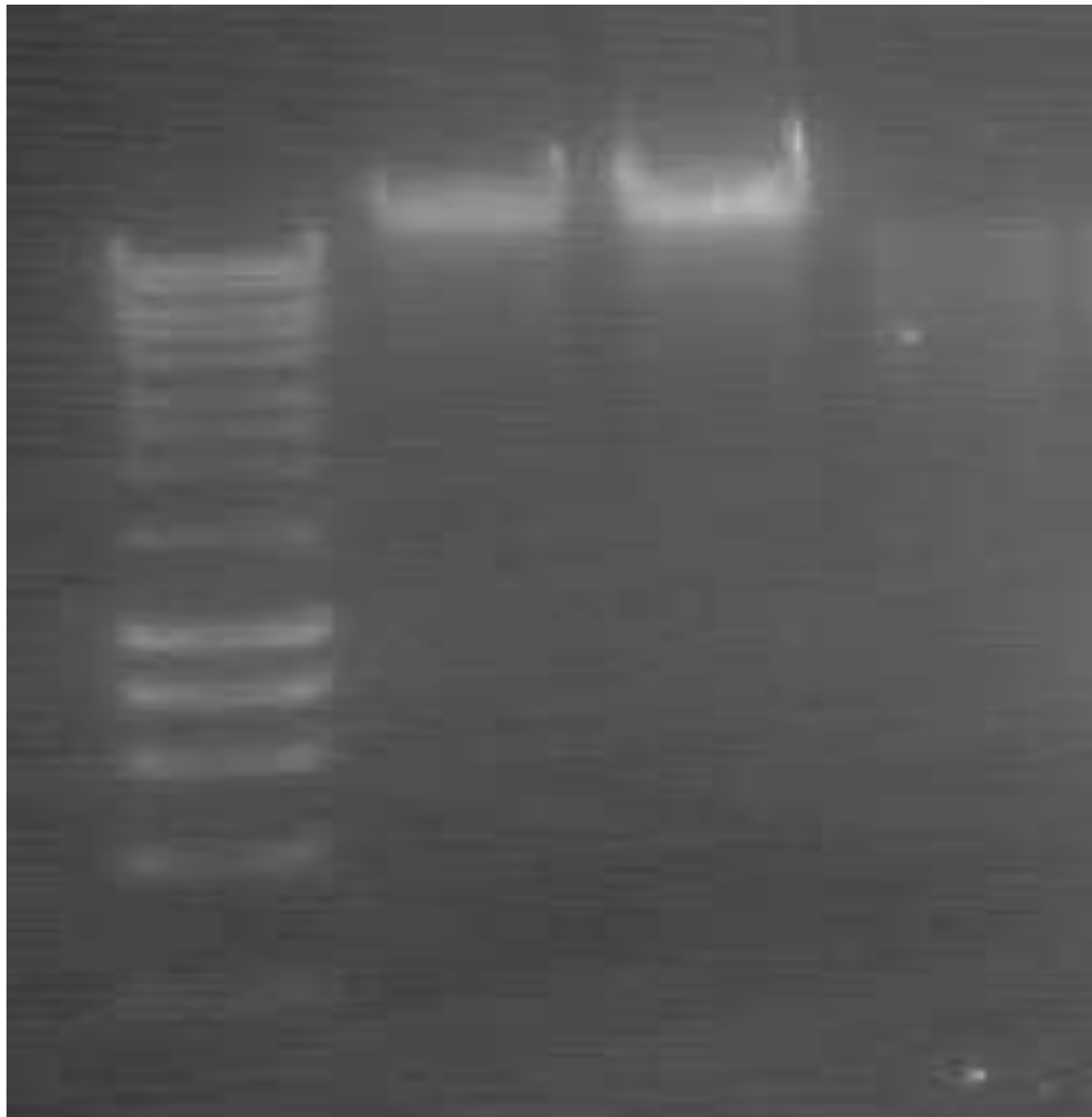

5

6

7

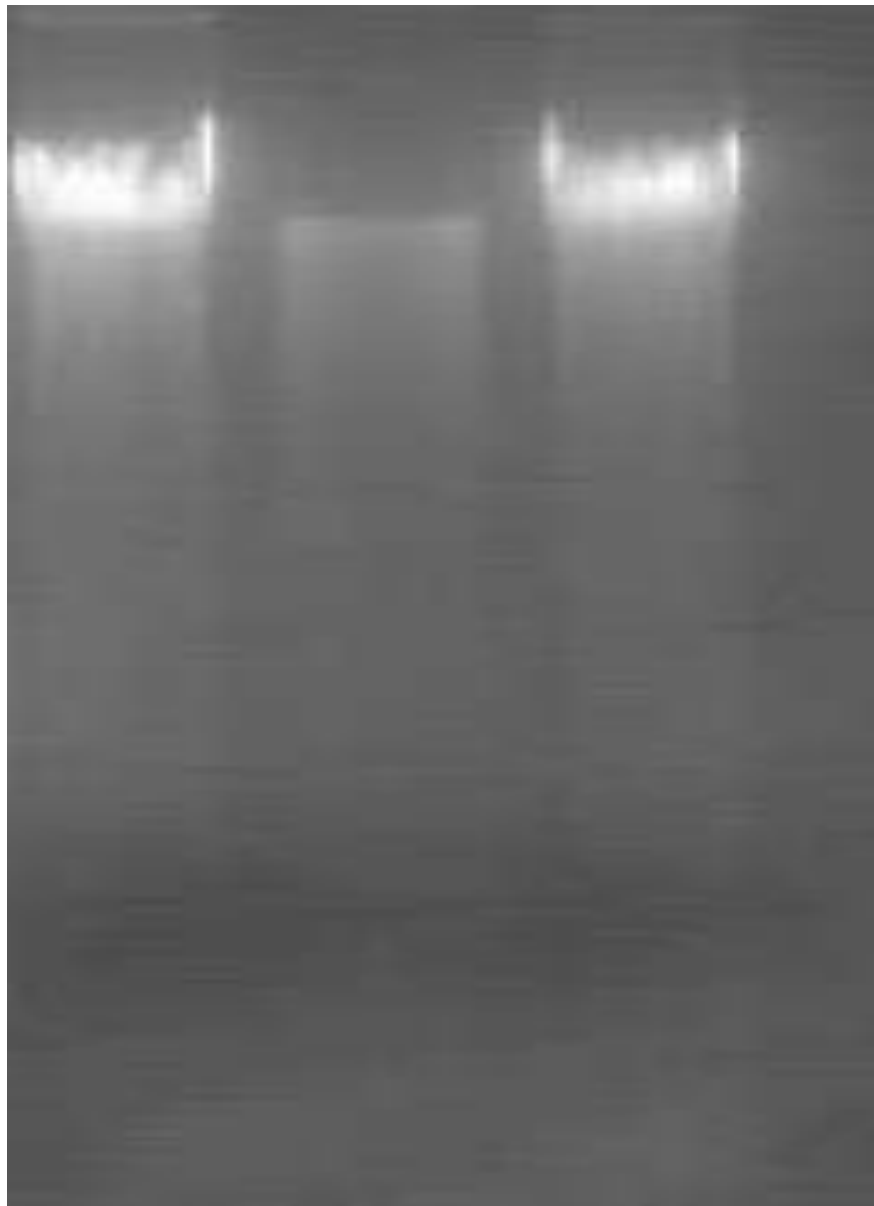

Supplement: Additional file 5: Figure S3. — Restriction analysis of B. longum genomic DNA. Restriction analysis of genomic DNA from B. longum subsp. longum NCIMB 8809 and B. longum subsp. longum CCUG 30698. Lane 1, molecular weight marker (Bioline). Lane 2: Unrestricted total DNA from B. longum subsp. longum NCIMB 8809, Lane 3 and 4 total B. longum subsp. longum NCIMB 8809 DNA restricted with lane 3 EcoRII and lane 4 PstI. Lane 5: Unrestricted total DNA from B. longum subsp. longum CCUG 30698, Lane 6 and 7 total B. longum subsp. longum CCUG 30698 DNA restricted with lane 6 EcoRII and lane 7 PstI. (PDF 93 kb) [file 12864_2015_1968_MOESM5_ESM.pdf]

A      1      2      3

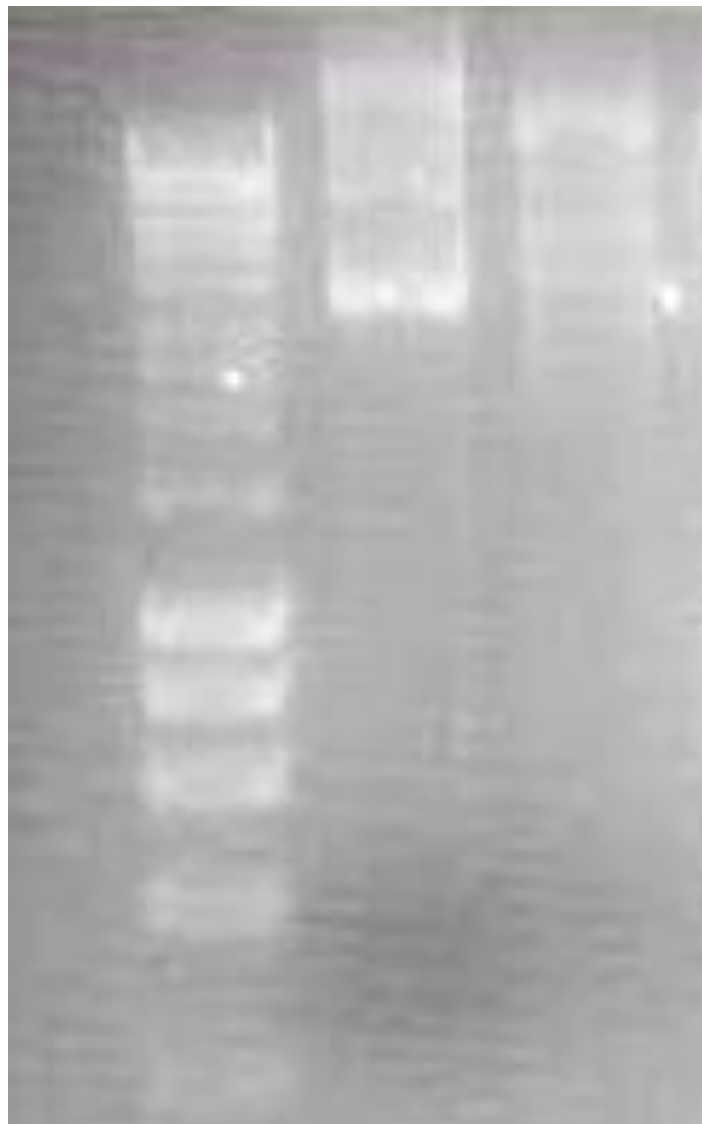

B      1      2      3

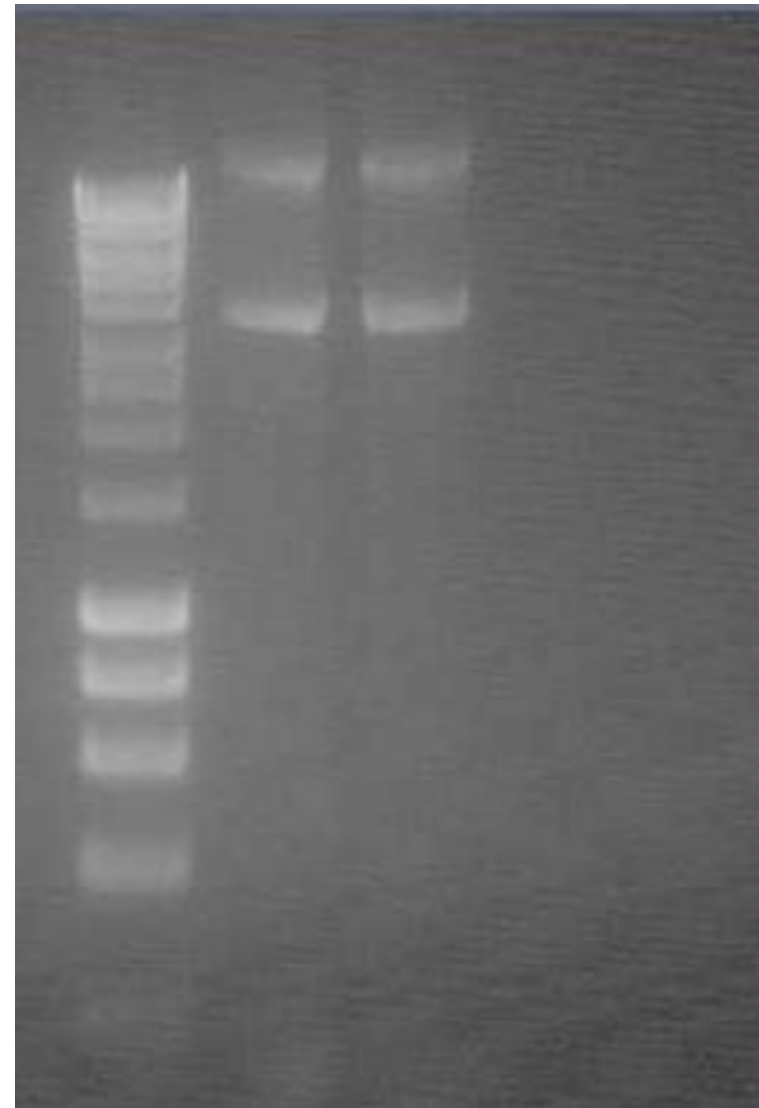

Supplement: Additional file 6: Figure S4. — Restriction analysis of plasmid DNA. a) Lane 1: molecular weight marker (Bioline). Lane 2 Unrestricted pAM5 plasmid DNA isolated from E. coli pNZEM-M.blmncII. Lane 3 pAM5 plasmid DNA isolated from E. coli pNZEM-M.blmncII and restricted with EcoRII. b) Lane 1: molecular weight marker (Bioline). Lane 2 unrestricted plasmid DNA pORI19-tetMod-ArfB isolated from E. coli pNZEM-M.blmncII. Lane 3 pORI19-tetMod-ArfB plasmid DNA isolated from E. coli pNZEM-M.blmncII and restricted with EcoRII. (PDF 102 kb) [file 12864_2015_1968_MOESM6_ESM.pdf]
